# Gamma transcranial alternating current stimulation increases segregation in the sensorimotor network

**DOI:** 10.3389/fpsyg.2026.1746459

**Published:** 2026-02-10

**Authors:** Antonio Cataneo, Marco Marino, Nicoletta Manzo, Cristina Scarpazza, Giorgio Arcara, Daniela Mapelli, Fabio Masina

**Affiliations:** 1 Department of General Psychology, University of Padova, Padova, Italy; 2 Movement Control and Neuroplasticity Research Group, KU Leuven, Leuven, Belgium; 3 IRCCS San Camillo Hospital, Venice, Italy; 4 Neurology Unit, San Filippo Neri Hospital ASL Roma 1, Rome, Italy; 5 Human Inspired Technology Research Centre, University of Padova, Padova, Italy

**Keywords:** brain oscillations, EEG, gamma oscillations, network connectivity, sensorimotor network, tACS

## Abstract

**Introduction:**

Transcranial alternating current stimulation (tACS) has emerged as a promising tool to modulate brain dynamics, especially in the context of motor recovery in clinical populations. Yet, its network-level effects on the sensorimotor (SM) functional organization have only been partially explored. In this study, we investigated whether gamma-frequency tACS can modulate functional connectivity and enhance segregation within the SM network, which is an index typically associated with better motor performance.

**Methods:**

In a within-subject, sham-controlled design, EEG was recorded before and after gamma tACS in 34 healthy subjects. Functional connectivity was quantified across three SM sub-networks, according to Yeo’s parcelation, in five frequency bands, including delta, theta, alpha, beta, and gamma, using intra- and inter-network connectivity (IntraNC and InterNC, respectively) measures. ANCOVA tests were performed on IntraNC and InterNC values for each frequency band, to compare the sham and real condition at post p-stimulation hase. The connectivity values of the pre-stimulation phase were used as a covariate, to account for state-dependent effects.

**Results:**

We did not find a significant interaction with time and condition. However, *post hoc* analyses showed significant interactions for IntraNC following real, but not sham, tACS (*p* = 0.029, FDR-corrected). Furthermore, we reported increased segregation post-tACS in specific SM sub-networks for alpha and beta frequency bands, primarily driven by enhanced IntraNC. No effects were observed in delta or theta frequency bands.

**Discussion:**

Albeit we did not find significant interactions of time and stimulation condition, additional analyses showed that gamma tACS might selectively modulate oscillatory dynamics within SM sub-networks, enhancing functional segregation in a frequency-specific manner. Given the roles of alpha/beta in sensorimotor integration and gamma in local processing, these effects might reflect more efficient neural communication. Our results support the potential of tACS as a neuromodulatory intervention to target dysfunctional network interactions in clinical populations.

## 1 Introduction

The sensorimotor (SM) network supports essential functions including voluntary movement, sensorimotor integration, and motor learning, and its functional architecture is tightly regulated through dynamic patterns of functional connectivity (Sahrizan et al., 2025). In this context, network segregation, which commonly reflects greater intra-network connectivity and/or lower inter-network connectivity, supports functional specialization across distributed brain systems (King et al., 2018; Samogin et al., 2022), and it is linked to an effective behavioral performance. In the aging brain, for example, several studies consistently reported lower connectivity within networks, including the SM network (Monteiro et al., 2019), and higher connectivity between them (King et al., 2018). Notably, the decreased segregation observed in older adults, which was mainly driven by increased inter-network connectivity, was associated with worse motor performance (King et al., 2018). Disruptions in the SM network organization have been observed in various neurological conditions, such as stroke and Parkinson’s disease, often manifesting as altered network segregation and impaired motor performance (Bonkhoff et al., 2020; D’Cruz et al., 2024). Higher SM network segregation, for example, has been seen to positively impact overall learning of writing skills in Parkinson’s disease, depending on disease duration (D’Cruz et al., 2024), and motor symptoms in severely, but not moderately, affected stroke patients (Bonkhoff et al., 2020), suggesting a differential impact of network segregation in relation to disease features and severity. To date, the potential use of non-invasive brain stimulation (NIBS) techniques to modulate this network has been only partially explored.

A useful NIBS technique that may potentially interfere with SM network is transcranial alternating current stimulation (tACS) (Antal et al., 2008; Marshall et al., 2006). tACS involves the direct delivery of alternating electrical currents to the scalp, which then passes through the scalp and skull to influence cortical neurons. This alternating current has a sinusoidal waveform in which the voltage gradually changes from positive to negative every half-cycle, flowing from a target electrode to a return electrode. The basic concept of this alternating current is to closely mimic the endogenous oscillatory pattern of electrophysiological activity in the brain (Elyamany et al., 2021), which can be detected using electroencephalography (EEG). Therefore, tACS has increasingly gained recognition as a method to modulate oscillatory brain activity and influence large-scale neural networks, becoming an extensively used NIBS technique in cognitive and motor neuroscience (Helfrich et al., 2014). However, the effects of tACS at the network level, particularly regarding the functional organization of the SM system, remain to be fully elucidated.

Electroencephalography enables precise detection of the brain’s characteristic oscillations. Evidence showed that different brain oscillations have been associated with different brain states (Kahana, 2006; Lee and Dan, 2012). Several studies showed that communication between distant cortical regions is associated with the synchronization of their brain oscillations (Bonnefond et al., 2017; Fries, 2005). Within the SM network, this synchronization facilitates the integration of information necessary to initiate, modulate, and execute movements. While alpha (8–13 Hz) and beta (13–30 Hz) oscillations are prominent in the sensorimotor cortex during the resting state (Salmelin and Hari, 1994), gamma oscillations (>30 Hz) predominate during movement execution and are linked to sensorimotor integration and fine control. In line with the role of gamma oscillations in facilitating local cortical synchronization by coordinating rapid rhythmic activity among neural populations (Moisa et al., 2016), gamma-frequency tACS may enhance segregation within the SM network by entraining and amplifying these endogenous gamma rhythms.

Gamma activity is known to support fine-grained motor coordination and sensorimotor integration, particularly within primary motor and somatosensory cortices (Miyaguchi et al., 2018). By entraining endogenous gamma rhythms, tACS may strengthen intra-network connections while reducing interference from unrelated or non-task-relevant regions, thus reinforcing functional boundaries and promoting network functional specialization, i.e., network segregation. This aligns with previous findings that associate gamma-band synchronization with higher local efficiency (Zhang et al., 2025) and, potentially, reduced global efficiency, both hallmarks of a segregated network architecture.

In parallel, it is plausible that alterations in segregation could also emerge in the alpha and beta frequency bands (Samogin et al., 2020, 2022), which play complementary roles in motor system processing. Alpha oscillations are often associated with functional inhibition and gating of sensory information, while beta activity is critically involved in maintaining the current motor state and mediating cortico-muscular coherence, as reflected by phase coupling between brain and muscle activity in the beta frequency band (Echeverria-Altuna et al., 2022). Modulation of these bands through tACS may impact long-range coordination and inhibitory control mechanisms, potentially influencing the balance between integration and segregation within motor-related circuits. Therefore, while gamma tACS may primarily drive local enhancements in segregation, changes in alpha and beta functional connectivity may reflect broader network-level adjustments in motor system functioning. Thus, functionally distinct roles of neural oscillations might shape the segregation of the SM network in response to non-invasive stimulation for specific frequency bands.

In this context, an increase in SM network segregation following gamma tACS may be interpreted as a positive, adaptive consequence of stimulation, leading to improved functional specialization and potentially reflecting enhanced performance in motor processes (Moisa et al., 2016). Given all these considerations, the potential ability of tACS in modulating brain rhythm in a frequency specific manner holds major theoretical and clinical implications. Clarifying the effects of tACS on SM network in healthy humans may provide useful information on its application in several neurological condition that underlie abnormal SM network functioning, that tACS may potentially restore.

In this study, we examined whether tACS alters the segregation of the SM network using EEG. Specifically, we aimed to explore how gamma tACS shapes functional connectivity in the SM network to explore the potential of EEG connectivity metrics as sensitive markers of network-level neuromodulation. To test this, we analyzed pre- and post-stimulation EEG in both real and sham tACS conditions, and we assessed changes in functional connectivity across delta, theta, alpha, beta, and gamma frequency bands.

## 2 Materials and methods

### 2.1 Ethical approval

The study was performed in agreement with the 1964 Helsinki Declaration and approved by the local ethics committee (Ethics Committee for Clinical Experimentation of the Province of Venice and the IRCCS San Camillo; Approval number: 2021.18). After being informed of the study’s objectives, methods, potential risks, and benefits, participants provided written informed consent.

### 2.2 Participants

A power analysis was conducted using G*Power software (Faul et al., 2007) to estimate the sample size. In the present study, assuming a small effect size (*d* = 0.25), an alpha level of 0.05, and a power of 0.9, the minimum required sample size is 30 participants. Thirty-four healthy participants (mean age = 28.4, SD ± 4.2; 22 females; mean education = 19.4, SD ± 1.7) with no history of neurological or psychiatric disease took part in the study. Participants were checked for tACS exclusion criteria (Antal et al., 2017). Thirty-two of 34 participants were right-handed, as assessed using Oldfield’s Edinburgh Handedness Inventory (Oldfield, 1971). This sample is the same as that used in a previous study with different hypotheses and objectives (Masina et al., 2025).

### 2.3 Procedure

**FIGURE 1:**
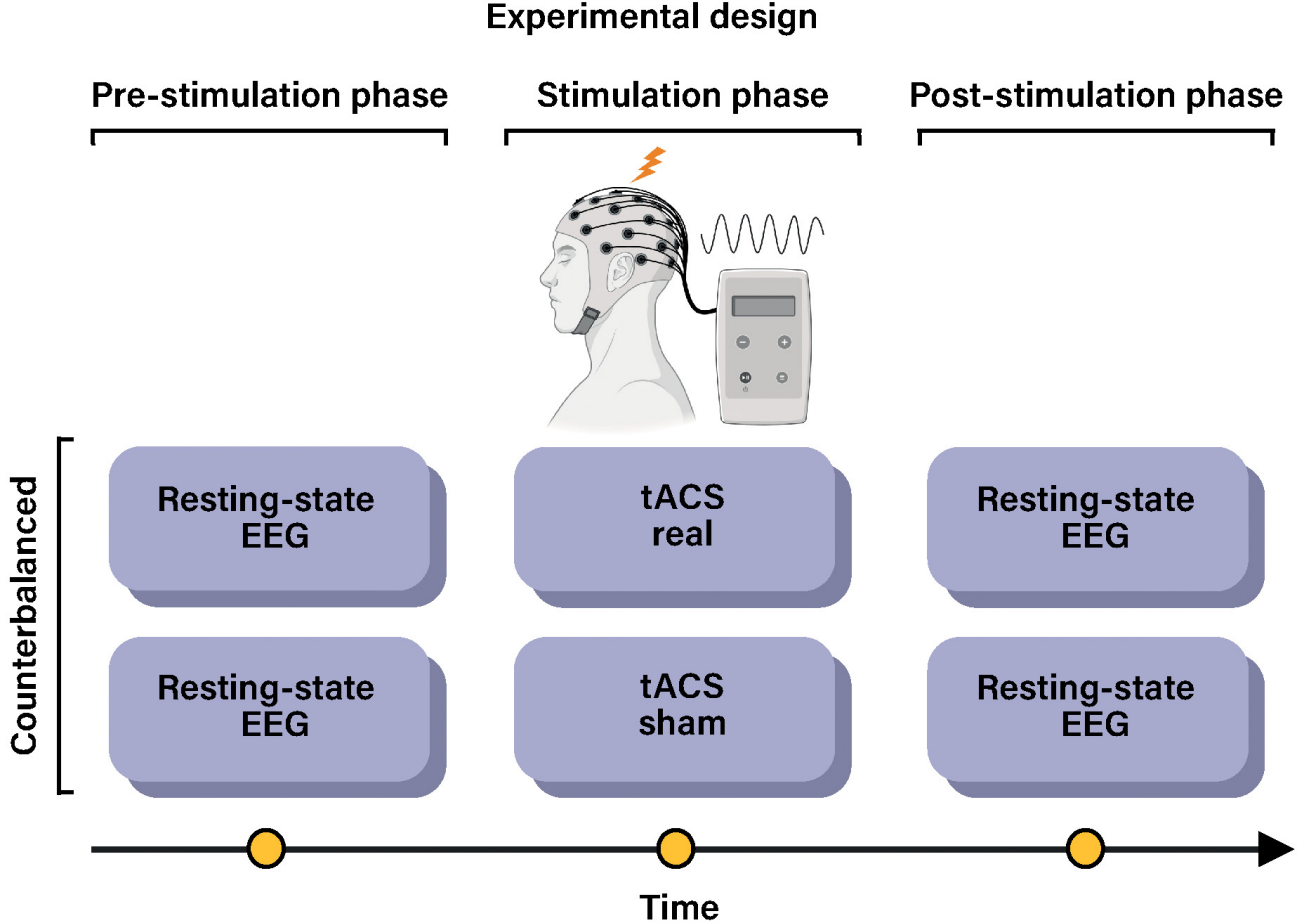
Experimental design and timeline of the gamma transcranial alternating current stimulation (tACS) procedure.

The study employed a paired, counterbalanced, and sham-controlled design comprising two separate stimulation sessions on different days, during which bilateral tACS was administered. This design ensured that each participant received both active and sham stimulation in a balanced order to control for placebo and order effects: each participant received both real and sham tACS in a counterbalanced order, with sessions spaced at least 5 days apart to minimize potential carry-over effects (Figure 1). Each session consisted of three phases. During the first phase (pre-stimulation phase), resting-state EEG was recorded for 5 min with eyes open. During the EEG recording, participants were asked to fixate a computer screen on a fixation point located 60 cm away. The second phase (stimulation phase) involved a 20-min tACS, either real or sham, during which participants remained seated and relaxed. Finally, the third phase (post-stimulation phase) replicated the same conditions as the pre-stimulation phase (i.e., resting state EEG).

### 2.4 Transcranial alternating current stimulation

**FIGURE 2:**
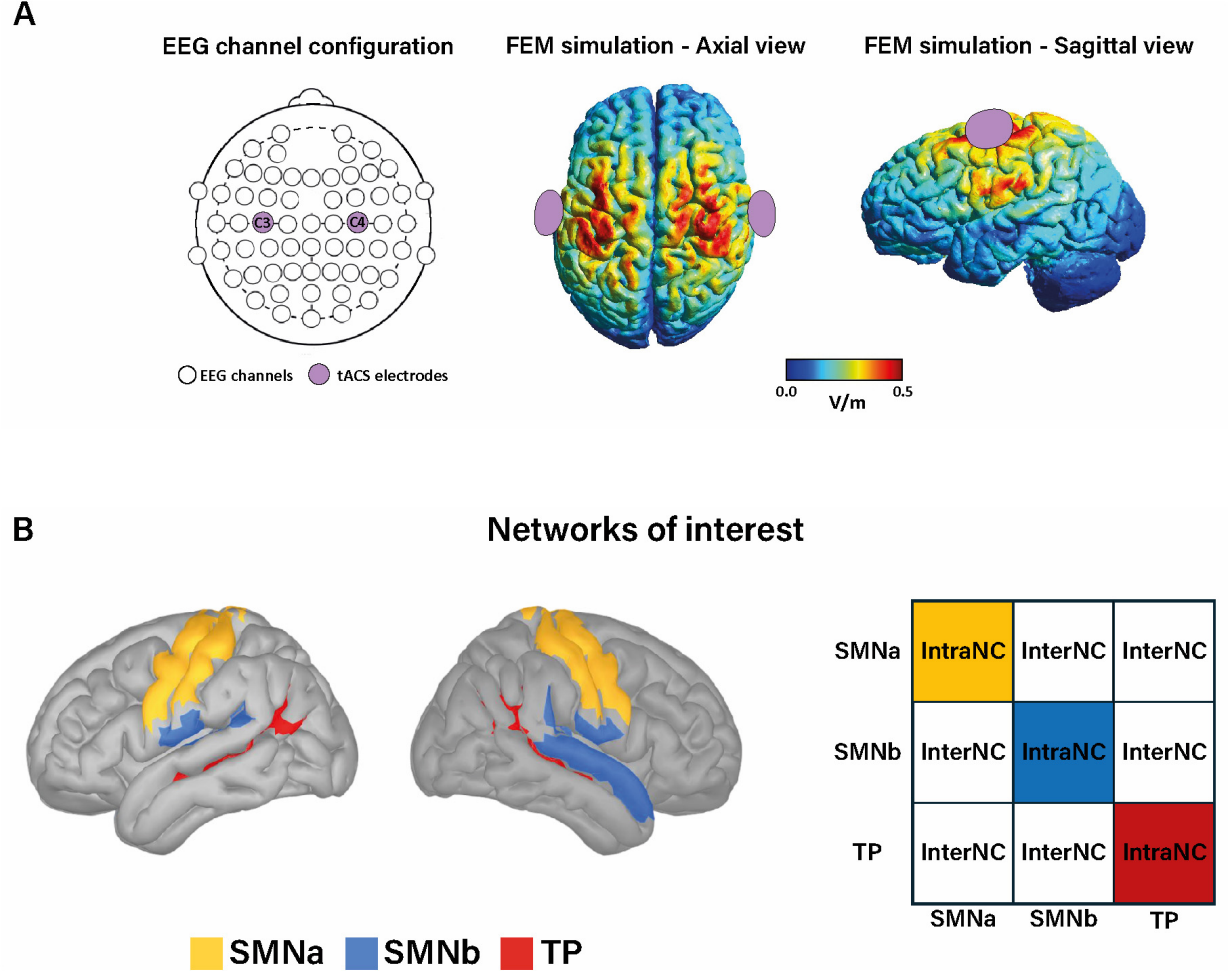
**(A)** In the left, the electroencephalography (EEG) channel configuration with 60 recording electrodes, with the two tACS electrodes positioned over C3 and C4, is displayed. In the middle and right, the finite element method (FEM) simulation of the tACS-induced electric field, estimated with SimNIBS (Thielscher et al., 2015), is shown for the axial view (middle) and the sagittal view (right). **(B)** In the left, the brain regions belonging to the SM network, according to the 17-network Yeo’s parcelation (Yeo et al., 2011), are displayed. For connectivity analysis, we considered the three sub- networks composing the SM network, including the SMNa (yellow), the SMNb (blue), and the temporoparietal (TP) (red). In the right, illustration of connectivity matrix showing the intra-network connectivity (IntraNC) values on the main diagonal, and the inter-network connectivity (InterNC) in the upper (lower) triangular matrix.

In the stimulation phase, administration of tACS followed the updated guidelines (Antal et al., 2017). Two circular saline-soaked sponge electrodes (surface = 8 cm^2^; current density: 0.25 mA/cm^2^) were placed over the SM regions. Before conducting the study, a finite element method simulation was performed using SimNIBS (Thielscher et al., 2015), a software designed to estimate the focality and intensity of the electric field for both transcranial magnetic stimulation and transcranial electrical stimulation (Busan et al., 2021). This simulation confirmed that the electric field generated by the current tACS configuration was mostly localized over the SM regions (Figure 2A). The stimulation current was delivered through a battery-driven stimulator (BrainStim, EMS Medical, Italy). The tACS electrodes were placed under the EEG cap at C4 and C3, according to the international 10–20 EEG system and corresponding to the right and left SM regions, respectively (Masina et al., 2022; Tang et al., 2023). The tACS electrodes remained in place for the entire session. The stimulation frequency was set to 40 Hz, with a peak-to-peak amplitude of 2 mA. Real and sham tACS included ramping-up and fading-out periods of 30 s each. The stimulation lasted 20 min, but during the sham session, no current was delivered except for 30 s at the beginning and the end of the 20 min of expected stimulation. At the end of each session, participants completed a questionnaire of stimulation-related sensations (Fertonani et al., 2015). Remarkably, participants were unable to distinguish between real and sham tACS [Session 1: Wald χ^2^(1) = 0.35, *p* = 0.551; Session 2: Wald χ^2^(1) = 0.28, *p* = 0.594].

### 2.5 EEG acquisition and data preprocessing

Participants were comfortably seated and asked to either relax or perform a task. During each session, EEG was recorded at a sampling rate of 1,000 Hz using the actiCHamp EEG amplifier, which was configured with 64 active EEG channels (Brain Products GmbH, Germany). All recordings were referenced to the AFz channel, while the ground was placed on FPz. The impedance of each EEG channel was kept lower than 5 KΩ throughout the recording. The EEG data were preprocessed offline with EEGLAB v2024.1 for Matlab R2021b (The Mathworks Natick, MA, United States). Raw EEG data were first processed by removing channels C3, C4, Iz, and an external channel, leaving 60 channels remaining. Channels C3 and C4 were removed because they were not connected during the recording; tACS electrodes were placed in their positions, making these channels non-functional for EEG analysis. The data were then down-sampled to 500 Hz. Next, a band-pass filter was applied between 0.1 and 70 Hz, followed by the removal of sinusoidal line noise at 50 Hz using the CleanLine plugin in EEGLAB (Delorme and Makeig, 2004)^1^. The continuous EEG signal was then visually inspected, and channels containing noisy signals were removed. Independent component analysis was applied to address any remaining artifacts, such as muscle activity and eye blinks. Each independent component was evaluated based on scalp distribution, frequency, timing, and amplitude (Chaumon et al., 2015). Rejected electrodes were reconstructed using EEGLAB spherical spline interpolation, ensuring continuity of the scalp topography. The EEG data were segmented into epochs, and the signal was re-referenced to the average of each epoch. Automated epoch rejection was applied, excluding epochs with amplitudes exceeding ±100 μV.

### 2.6 Source estimation and connectivity analysis

For source analysis, we used the standardized low-resolution electromagnetic tomography (sLORETA) method, as implemented in Brainstorm software (Tadel et al., 2011). The head model was computed using the ICBM 152 MRI template anatomy using OpenMEEG BEM (Gramfort et al., 2010). The brain was parcelated into bilateral regions of interest (ROIs) according to the Destrieux Atlas (Destrieux et al., 2010). According to Yeo’s parcelation, Destrieux regions were grouped into 17 networks of interest (Yeo et al., 2011). We extracted the Amplitude Envelope Correlation (AEC) measure to assess modulations in functional connectivity within the SM network, by including in our analyses three sub-networks, belonging to the SM network according to Yeo, i.e., temporoparietal (TP) and SM networks (SMNa and SMNb) (Figure 2B). AEC-based connectivity values were extracted from the resting-state sessions (pre and post-stimulation phase) and averaged across five canonical frequency bands: delta (1–4 Hz), theta (4–8 Hz) alpha (8–13 Hz), beta (13–30 Hz), gamma (30–50 Hz). We examined the connectivity between pairs of SM regions, for each frequency band. In particular, we defined intra-network connectivity (IntraNC) as the average connectivity between pairs of ROIs within a specific SM sub-network. Similarly, inter-network connectivity (InterNC) was calculated as the average connectivity between all the possible pairs of ROIs belonging to different SM sub-networks (Newton et al., 2011; Samogin et al., 2020; Figure 2B).

To investigate the overall tACS induced effects on network connectivity, we performed a 2 × 2 repeated-measures ANCOVA tests on IntraNC and InterNC values with the factors CONDITION (two levels: sham, real) and TIME (two levels: pre-stimulation, post-stimulation). These tests were run separately for each frequency band and allowed the comparison of the sham condition with the real condition at post-stimulation phase. To this end, we used the connectivity values of the pre-stimulation phase as a covariate, to account for state-dependent effects (Masina et al., 2021, 2022).

To further characterize any condition-specific effects, we conducted, separately for the sham and real conditions, as a *post hoc* exploratory analysis, a 3-way repeated-measures ANOVA tests on IntraNC and InterNC values, with TIME (two levels: pre-stimulation, post-stimulation), FREQUENCY (five levels: delta, theta, alpha, beta, gamma), and NETWORK (three levels, corresponding to the functional networks considered) as within-subject factors. We finally performed a two-tailed paired *t*-test on IntraNC and InterNC values, for each frequency band and each SM sub-networks, to assess network segregation for each considered time (pre and post) and condition (sham and real). We used the false discovery rate (FDR) method to account for multiple comparisons, and the significance level was set to *p* < 0.01.

## 3 Results

The preliminary overall analysis using ANCOVA tests revealed no significant changes across the four experimental conditions (sham pre-stimulation, sham post-stimulation, real pre-stimulation, and real post-stimulation) either for TP or SM networks, in any of the considered frequency bands, suggesting similar changes over time across groups (Supplementary Figure 1). Given the precise expectations of our research, we further proceeded with exploratory analyses investigating the tACS effects, separately for each stimulation condition. These ANOVA tests, performed separately for each stimulation condition (sham and real), showed significant interactions. In the Real stimulation, the ANOVA test revealed significant interactions (*p* = 0.029, FDR-corrected) between time, frequency, and network, for the IntraNC, but not for the InterNC (*p* = 0.7508, FDR-corrected), suggesting that pre-to-post connectivity changes occur across frequency bands and networks when considering more local interactions, rather than whole-brain. On the other hand, in the sham stimulation, no significant interactions were found either for the IntraNC (*p* = 0.2345, FDR-corrected) or InterNC (*p* = 0.4503, FDR-corrected), showing that the sham did not lead to relevant changes in connectivity.

**FIGURE 3:**
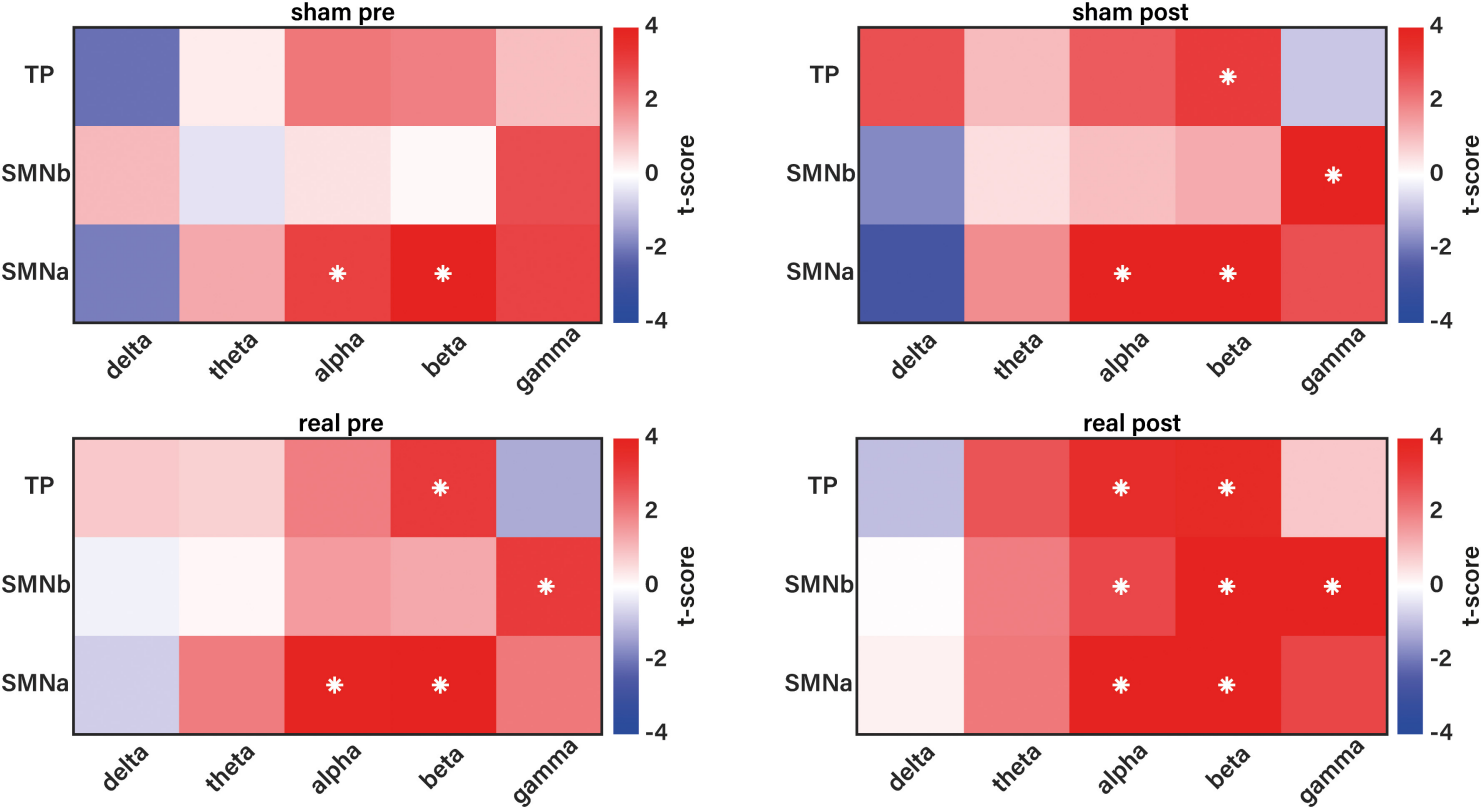
Comparison between intra- and inter-network connectivity (IntraNC and InterNC, respectively), for each pair of networks in the five frequency bands (delta, theta, alpha, beta and gamma). For each condition and time, we used a two-tailed paired *t*-test to compare IntraNC and InterNC. The comparisons related to the sham condition (pre and post stimulation) are at the top, and the values related to the real condition (pre and post stimulation) are at the bottom. The asterisks indicate significant effects (*p* < 0.01, FDR-corrected).

When assessing network segregation (Figure 3), we found that, at baseline, IntraNC was larger than InterNC at baseline for TP in the beta frequency band, for SMNb in the gamma frequency band and for SMNa in the alpha and beta frequency bands. This trend overall applied for both the real and sham conditions in the pre-stimulation phase.

Following the stimulation phase, for the real stimulation condition, we reported increased segregation for different networks in different frequency bands, including TP in the alpha frequency band (*t* = 3.58, *p* = 0.002) and SMNb in the alpha (*t* = 2.93, *p* = 0.009) and beta (*t* = 4.1, *p* = 0.0006) frequency bands. This is likely to be driven by the increased IntraNC, as emerged from the previously reported ANOVA results for the real stimulation condition. We also reported fewer alterations following the sham stimulation condition, even if these alterations seem to be time-dependent, resulting in a strengthening of the segregation pattern reported at baseline, rather than affecting other regions in other frequency bands. Overall, these results indicate that gamma tACS can be used to modulate network connectivity. In particular, the real stimulation leads to increased network segregation compared to sham stimulation, especially in the alpha and beta frequency bands across SM sub-networks. Following gamma tACS, no modulation was reported in the delta and theta frequency bands.

## 4 Discussion

In this study, we investigated the effects of gamma tACS on functional connectivity within the SM network. Our findings demonstrate that gamma tACS can modulate brain network dynamics in a frequency- and network-specific manner, particularly enhancing functional segregation across SM sub-networks (SMNa, SMNb, TP). Following brain stimulation, we observed increased functional segregation for different SM sub-networks in different frequency bands, including alpha, beta, and gamma frequency bands. This latter result suggests that gamma tACS can modulate connectivity patterns, promoting more functionally specialized processing. Indeed, increased segregation, which could result from higher IntraNC and/or lower InterNC, typically reflects more specialized and efficient information processing (King et al., 2018; Samogin et al., 2022). This shift in the SM network may support task demands involving refined motor control or focused processing of somatosensory stimuli.

Although our ANCOVA tests did not show any significant interactions, condition-specific analyses revealed more nuanced effects (Figure 3). Notably, in the real stimulation condition, we observed a significant Time × Frequency × Network interaction for IntraNC, but not for InterNC. This suggests that tACS primarily could modulate local intra-network neural communication, rather than global inter-network interactions. These changes were not present during sham stimulation, reinforcing that the effects are specific to the real stimulation. Critically, *post hoc* comparisons indicated increased functional segregation in different SM subnetworks, such as TP and SMNb, following gamma tACS. This was particularly evident in the alpha and beta frequency bands, with significant increases in segregation for TP and SMNb in both alpha and beta frequency bands. Importantly, these effects appear to be driven primarily by increases in IntraNC, rather than by decreases in InterNC, consistent with the view that tACS promotes more functionally specialized processing within targeted networks.

From a frequency-domain perspective, gamma oscillations are often associated with local cortical processing and short-range neural communication (Ray and Maunsell, 2015) and are implicated in motor execution and sensorimotor integration (Ulloa, 2022). In contrast, alpha and beta frequency bands are known to support top-down inhibitory control (Hwang et al., 2014; Pascucci et al., 2025), movement gating (Tzagarakis et al., 2010), and long-range interactions within sensorimotor pathways (Ulloa, 2022). The observed frequency-specific modulations are consistent with previous research regarding the functional roles of these oscillatory frequency bands in the sensorimotor system. Gamma stimulation with tACS can lead to cross frequency effects (Venugopal et al., 2025). In particular, the entrainment of alpha oscillations following gamma tACS aligns with established findings indicating that tACS can synchronize neuronal activity to specific frequency bands, such as alpha, through entrainment mechanisms (Helfrich et al., 2014). Indeed, the observed increase in network segregation across these frequency bands suggests that gamma tACS may enhance both local processing (via gamma) and integration within broader sensorimotor networks (via alpha and beta), potentially optimizing the balance between local specialization and inter-regional coordination required for sensorimotor functioning. The observation of baseline segregation patterns, with higher IntraNC than InterNC in specific networks and frequency bands suggests that tACS may act to reorganize the SM system frequency- and network-specific manner. Importantly, these effects were absent or minimal in the Sham condition, which primarily showed time-dependent reinforcement of baseline patterns, rather than the emergence of new segregation patterns. Taken together, these results support the hypothesis that gamma tACS can induce state-dependent modulation of brain connectivity (Silvanto et al., 2008; Bergmann, 2018). It is well-established that the effects of NIBS are influenced by the brain’s functional state at the time of stimulation, which reflects both ongoing neural dynamics and the immediate history of brain activity (Bergmann, 2018). Traditionally, operationalizations of brain states have encompassed a wide range of measures, from affective or cognitive states (Di Rosa et al., 2024; Schutter et al., 2023) to transient oscillatory features such as frequency, amplitude, and phase (Masina et al., 2021, 2022; Zrenner et al., 2020). Our findings indicate that tACS-induced functional changes are shaped by the pre-stimulation functional configuration of the targeted networks, consistent with emerging views on the importance of individualized stimulation protocols and state-informed stimulation approaches.

Previous studies have shown that tACS can entrain endogenous oscillations in a frequency-specific manner (Wischnewski et al., 2023), thus facilitating inter-areal synchronization and the transmission of information between distant cortical regions. In this context, our observations of enhanced network segregation, especially, in the alpha and beta frequency bands, following gamma tACS, is particularly relevant, as these rhythms have been associated with sensory gating mechanisms and motor state maintenance (Foxe and Snyder, 2011; Tzagarakis et al., 2010), respectively. In clinical contexts, such modulation may have promising therapeutic implications for neurological conditions characterized by disruptions in large-scale network dynamics, linked to altered network segregation. Studies in patients with stroke showed that both intra- and inter-hemispheric connectivity in the SM network are abnormal (Grefkes and Fink, 2011) in acute and chronic phases. Interestingly, these abnormalities correlate with impaired motor recovery and fatigue (Sahrizan et al., 2025). Based on this evidence, the tACS-induced modulation of network dynamics in patients with stroke, combined with rehabilitations programs, may potentially improve motor recovery in these patients. Similarly, a reduced IntraNC within the SM network has been largely described in patients with Parkinson’s disease (PD) (Caspers et al., 2021). In particular, PD patients with greater motor deficits showed significant disconnection within the SM network, whereas patients with non-motor deficits exhibited reduced FC in an extended subnetwork, mainly involving the SM areas (Kicik et al., 2025). Our findings suggest that tACS applied over SM areas can contribute to increase network segregation. This might have beneficial effects in the clinical context, by restoring, for example, impaired connectivity in PD patients, increasing frequency-specific network segregation within SM circuits. It is to note that, according to available knowledge, the interference with this functional imbalance in network dynamics may potentially have a clinical impact, improving motor symptoms in PD.

This study presents several limitations that warrant consideration. First and foremost, our preliminary analysis using ANCOVA tests did not show a significant interaction, and as such all results must be taken as exploratory with a strong need of collection of further evidence. Second, from a methodological point of view, although the EEG electrode density employed was adequate for the current analyses, future studies would benefit from the use of high-density EEG systems (e.g., 128 or 256 channels), which offer improved spatial resolution for source localization and enhanced reliability in connectivity assessments (Marino and Mantini, 2024). Third, while power envelope-based metrics, such as AEC, provided valuable insights into large-scale functional interactions, incorporating alternative measures of brain connectivity, such as phase-based metrics, could yield a more comprehensive characterization of inter-regional neural dynamics. Fourth, the tACS protocol could be further individualized with respect to stimulation parameters, including frequency, intensity, and duration, to enhance both its efficacy and specificity. Finally, although the observed modulation of connectivity is promising, the behavioral relevance of these neurophysiological changes still remains to be fully clarified. In the present study, participants performed a behavioral task before and after tACS. Nevertheless, this task was designed for purposes other than the ones of the present study, as described in Masina et al. (2025), and resulted in ceiling effects. Accordingly, no performance differences were observed between the real and sham conditions. Future studies could link changes in network segregation to performance by including a more complex task that could avoid ceiling effects. Further research is necessary to determine whether such connectivity alterations translate into stable and generalizable improvements in motor performance. Overall, future studies should aim to integrate more advanced methodological approaches with personalized stimulation protocols to improve the interpretability, robustness, and translational value of these findings.

In conclusion, gamma tACS appears to boost the segregation of SM sub-networks within the alpha and beta frequency bands, suggesting enhanced functional specialization of this cortical system. This supports the hypothesis that gamma-frequency stimulation can induce targeted neuromodulatory effects within specific cortical circuits. Thus, these results contribute to advancing our understanding of frequency-specific brain stimulation and may pave the way for personalized rehabilitation approaches in movement disorders.

## Data Availability

The raw data supporting the conclusions of this article will be made available by the authors, without undue reservation.

## Footnotes

1
https://www.nitrc.org/projects/cleanline/
