# Supplementary material for: Gamma transcranial alternating current stimulation increases segregation in the sensorimotor network
Source: Front Psychol. 2026 Feb 10;17:1746459. doi: 10.3389/fpsyg.2026.1746459 (PMC12929117; doi:10.3389/fpsyg.2026.1746459)
Supplement: Supplementary file 1 [file Table_1.docx]

**Gamma transcranial alternating current stimulation increases segregation in the sensorimotor network**

**Supplementary material**


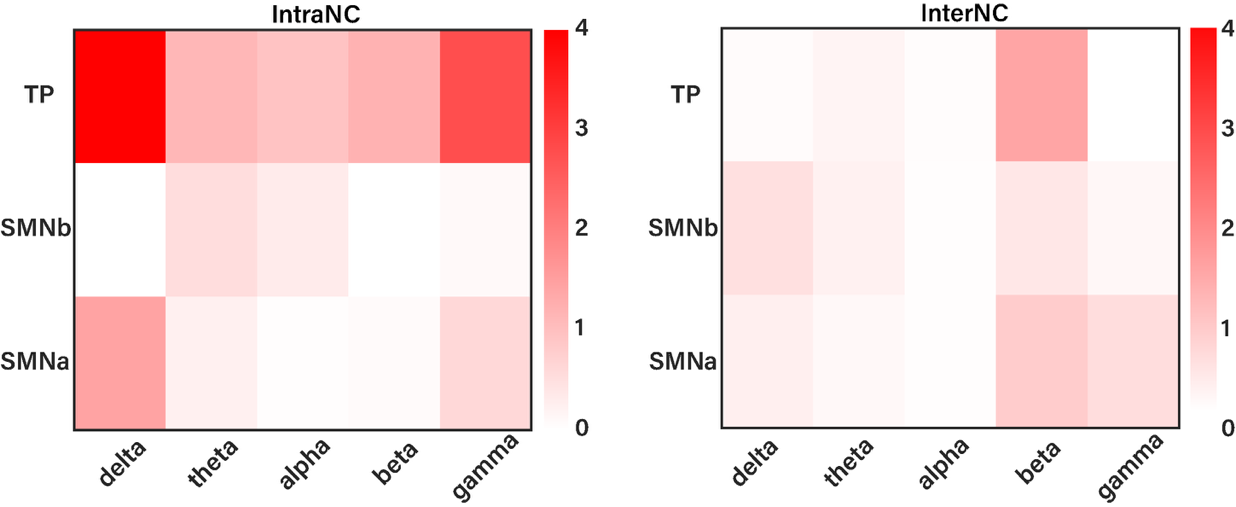


**Figure S1.** *Results of the 2 × 2 repeated-measures ANCOVA tests on IntraNC (left) and InterNC (right) values with the factors CONDITION (two levels: sham, real) and TIME (two levels: pre-stimulation, post-stimulation). These tests were run separately for each frequency band and allowed the comparison of the sham condition with the real condition at post-stimulation phase.*
